# Long-Term Clinical Efficacy, Safety, and Polypharmacy Simplification of Levodopa–Entacapone–Carbidopa Intestinal Gel in Advanced Parkinson’s Disease: An 18-Month Longitudinal Study

**DOI:** 10.3390/jcm15187319

**Published:** 2026-09-21

**Authors:** Károly Orbán-Kis, Szabolcs Szatmári, Viorelia Adelina Constantin, Róbert Máté Szász, Simona Bățagă, János Szederjesi, Marius Ciorba, Előd Ernő Nagy, Radu Mircea Neagoe, Krisztina Kelemen, Konrád Bíró, Crăciun Ciorba Nicoleta, István Mihály, Attila Frigy, József Attila Szász

**Affiliations:** 1 George Emil Palade University of Medicine, Pharmacy, Science and Technology of Târgu Mureș, 540142 Târgu Mureș, Romania; karoly.orban-kis@umfst.ro (K.O.-K.); szasz.robert-mate.20@stud.umfst.ro (R.M.S.); simona.bataga@umfst.ro (S.B.); janos.szederjesi@umfst.ro (J.S.); marius.ciorba@umfst.ro (M.C.); elod.nagy@umfst.ro (E.E.N.); radu.neagoe@umfst.ro (R.M.N.); krisztina.kelemen@umfst.ro (K.K.); nicoleta.craciun-ciorba@umfst.ro (C.C.N.); attila.frigy@umfst.ro (A.F.); jozsef.szasz@umfst.ro (J.A.S.); 2 2nd Clinic of Neurology, Mures County Emergency Clinical Hospital, 540142 Târgu Mureș, Romania; vioreliaconstantin@yahoo.com (V.A.C.); biro.konrad1999@gmail.com (K.B.); 3 Department of Gastroenterology, Mures County Emergency Clinical Hospital, 540142 Târgu Mureș, Romania; 4 Laboratory of Medical Analysis, Clinical County Hospital Mures, 540142 Târgu Mureș, Romania; 5 2nd Clinic of Surgery, Mures County Emergency Clinical Hospital, 540142 Târgu Mureș, Romania; 6 Department of Neurology, Emergency County Hospital, 530173 Miercurea Ciuc, Romania; 7 Department of Internal Medicine IV, Clinical County Hospital Mures, 540142 Târgu Mureș, Romania

**Keywords:** advanced Parkinson’s disease, levodopa–entacapone–carbidopa intestinal gel, device-aided therapy, motor fluctuations, dyskinesia, long-term monitoring

## Abstract

**Background:** Advanced Parkinson’s disease (aPD) is characterized by motor fluctuations, unpredictable oral absorption, and severe dyskinesias. Levodopa–entacapone–carbidopa intestinal gel (LECIG) is designed to provide dopaminergic stimulation and optimize levodopa bioavailability. We evaluated 18-month clinical outcomes, motor stabilization, disease burden, and oral medication simplification in a real-world cohort transitioning to LECIG. **Methods:** This retrospective longitudinal study included 41 patients with medication-refractory aPD. Patients underwent nasojejunal titration (mean 5.58 ± 1.50 days), followed by percutaneous endoscopic gastrojejunostomy. Assessments were performed at baseline, initiation, and 6, 12, and 18 months. Primary endpoints were daily OFF time, dyskinesia duration, and non-linear fluctuations. Disease burden was analyzed using Linear Mixed-Effects Models and medication changes using McNemar tests and paired *t*-tests. **Results:** Patients (56.1% male; mean age 65.39 ± 8.32 years; disease duration 10.46 ± 4.23 years) demonstrated significant and sustained clinical improvements. For the 38 patients evaluable at 18 months, daily OFF time decreased from 4.67 ± 0.79 h to 1.34 ± 0.40 at initiation and 1.55 ± 0.69 at 18 months (*p* < 0.0001; 66.8% reduction). Severe peak-dose dyskinesia was absent after initiation (2.17 ± 1.00 to 0.00 h/day; *p* < 0.001), whereas early morning akinesia decreased from 78.0% to 36.6% and freezing of gait from 56.1% to 31.6%. Total Clinical Burden Score decreased from 9.33 to 3.49 (−62.6%). Dopamine agonist use decreased from 80.5% to 42.1% at 18 months (McNemar *p* < 0.0001), while adjunct medications decreased from 1.73 to 1.39/patient (*p* = 0.0284). Adverse events were predominantly mild-to-moderate. No patient discontinued therapy because of an adverse event or hardware failure; one patient discontinued LECIG by personal choice. Two additional patients died from sudden cardiac arrest during follow-up, both considered unrelated to LECIG therapy or pump hardware. At 18 months, 38 of 41 patients remained evaluable. **Conclusions:** In this observational cohort, LECIG initiation was followed by sustained reductions in motor complications, OFF time, and clinical burden over 18 months. Treatment was also associated with a reduction in dopamine agonist use and overall adjunctive medication burden. The favorable tolerability and low discontinuation rate observed support further evaluation of LECIG as an advanced treatment pathway. However, pre-switch LEDD calculations may support initial pump programming but should not be considered definitive predictors of individualized maintenance settings; inpatient clinical titration remains necessary.

## 1. Introduction

Parkinson’s disease (PD) is a progressive neurodegenerative disorder primarily characterized by the loss of dopaminergic neurons [1]. While oral administration of levodopa (LD) combined with peripheral dopa-decarboxylase inhibitors remains the gold standard for symptomatic management, its long-term utility is severely challenged by disease progression [2]. Within 5 to 10 years of initiating therapy, more than 50% of patients develop disabling motor fluctuations and dyskinesias. These motor complications arise from a combination of narrowing therapeutic windows, a shortened plasma half-life of oral levodopa, and irregular gastrointestinal transport—frequently exacerbated by delayed gastric emptying and erratic intestinal absorption [3,4]. Under standard oral regimens, the intermittent, pulsatile stimulation of central postsynaptic dopamine receptors leads to maladaptive plastic changes in the striatal neural circuitry, manifesting clinically as wearing-OFF phenomena, peak-dose dyskinesias, and sudden OFF states [5,6,7,8]. To mitigate these fluctuations, clinicians routinely introduce complex oral and transdermal adjunctive therapies, including dopamine agonists (DAs), monoamine oxidase B inhibitors (MAO-Bi), and catechol-O-methyltransferase inhibitors (COMTi). However, as advanced Parkinson’s disease (aPD) develops, these multidrug combinations become increasingly ineffective, increase pill burden, and significantly elevate the risk of severe side effects, such as impulse control disorders, hallucinations, and orthostatic hypotension [9,10].

To address the limitations of conventional oral therapies, continuous drug delivery modalities, also called device-aided therapies (DATs), have emerged as transformative interventions [11]. Continuous intrajejunal levodopa infusion circumvents gastric dysmotility by delivering the medication directly into the site of maximal absorption in the proximal small intestine [12,13]. This approach minimizes plasma concentration volatility and establishes continuous dopaminergic stimulation, mimicking a more physiologic state [8,14,15]. Levodopa-carbidopa intestinal gel (LCIG) has long been established as an effective DAT, demonstrated to reduce daily OFF time by 2 to 4 h and significantly reduce dyskinesia burden [16,17].

The therapeutic framework has recently evolved with the introduction of levodopa–entacapone–carbidopa intestinal gel (LECIG) [18,19]. This formulation also integrates entacapone, a potent peripherally acting COMTi, which significantly increases the area under the plasma concentration-time curve (AUC) of levodopa [20,21], expanding its central bioavailability by 20% to 25% compared to standard LCIG [22]. This biochemical synergy permits an approximate 20% to 35% reduction in the total daily continuous levodopa dose while maintaining highly stable, therapeutically effective plasma concentrations [22,23]. Additionally, LECIG is administered via a specialized micro-infusion pump that is smaller and lighter than standard LCIG hardware, directly addressing patient compliance and physical handling challenges [18].

Furthermore, continuous levodopa exposure is often limited by a high incidence of peripheral neuropathy [24,25], which is strongly correlated with elevated cumulative doses and accumulation of the toxic metabolite homocysteine [18,26]. Because entacapone blocks the methylation pathway responsible for generating homocysteine from levodopa, it may exert a long-term protective effect against large-fiber and small-fiber polyneuropathy, representing a distinct advantage over traditional LCIG formulations [23,26].

While short-term studies have established the immediate bioequivalence and safety of LECIG [22], real-world data evaluating multi-milestone long-term outcomes remain sparse [23,27]. Tertiary neuro-movement specialist centers, such as our clinic in Târgu Mureș, Romania, possess over fifteen years of experience in managing intrajejunal gel infusions [28,29]. Unfortunately, in our regional clinical environment, patient access to deep brain stimulation (DBS) or subcutaneous apomorphine pumps is highly constrained by structural and socioeconomic limitations, positioning enteral gel infusion as the primary available advanced intervention [28,30]. Consequently, aPD cohorts in our region frequently present with exceptionally high baseline symptom severity and significant adjunctive polypharmacy, as clinicians maximize oral regimens before introducing invasive procedures [28,30].

This study presents an 18-month longitudinal evaluation of a consecutive cohort of 41 aPD patients de novo transitioning to LECIG therapy. We analyze the acute effects of inpatient titration and follow patients across multiple long-term milestones. Our objective was to comprehensively quantify the sustained efficacy of LECIG on continuous motor parameters, track the evolution of motor complications, map the structured simplification of adjunctive polypharmacy over time, and evaluate long-term safety and attrition profile in this cohort.

## 2. Materials and Methods

### 2.1. Patient Selection and Ethical Considerations

This retrospective, single-center longitudinal study evaluated 41 consecutive patients with advanced, medication-refractory PD who were initiated de novo on LECIG infusion therapy between December 2021 and June 2026 at the 2nd Clinic of Neurology within the Mureș County Emergency Clinical Hospital in Târgu Mureș, Romania. Patient eligibility was rigorously defined using validated consensus criteria—the Antonini et al. Delphi panel definition [31], the CEPA study’s advanced PD criteria [32], and the 5-2-1 screening tool [33]—as well as applicable national guidelines [34]. All patients underwent comprehensive baseline screening by a specialized multidisciplinary movement disorders team comprising a neurologist, a psychiatrist, a gastroenterologist, and a clinical psychologist.

Patients were treated and evaluated under a prospective clinical protocol approved by the Ethics Committee of the George Emil Palade University of Medicine, Pharmacy, Science and Technology of Târgu Mureș (UMFSTGEP; Approval Number: 94/19 May 2017). All participants provided written informed consent at hospital admission and prior to clinical enrollment. Because the present study is a retrospective, secondary analysis of routinely collected data obtained under this previously approved protocol—with no additional interventions or diagnostic procedures performed—the requirement for a separate, subsequent ethical approval was waived by the institutional Ethics Committee in accordance with institutional policy. The study was conducted in full accordance with the principles of the Declaration of Helsinki.

### 2.2. Inpatient Titration Phase and Dosing Protocol

In accordance with national regulatory guidelines [34], all patients were managed within a mandatory continuous inpatient framework divided into two discrete steps: a nasojejunal testing phase and a post-percutaneous endoscopic gastrojejunostomy (PEG-J) optimization phase. A temporary nasojejunal tube was placed into the proximal small intestine without requiring fluoroscopic or radiological control. Over a baseline testing period, LECIG gel was infused continuously using the specialized micro-infusion pump to demonstrate immediate therapeutic responsiveness and establish specific tolerability.

The initial dosing structure was mathematically derived from the patient’s pre-switch oral Levodopa Daily Equivalent Dose (LEDD), calculated via established clinical algorithms [18,35]. Following stabilization on the temporary probe, a definitive PEG-J system was surgically inserted by the gastroenterology team [36,37]. The final dosing regimen delivered at discharge comprised three elements: a morning bolus dose, a customized hourly continuous infusion rate, and patient-activated extra rescue doses [38].

### 2.3. Clinical Endpoints and Longitudinal Monitoring

Standardized clinical data were extracted from prospective registry logs captured at five systematic longitudinal milestones: baseline, LECIG initiation, 6 months, 12 months, and 18 months. Motor endpoints included mean daily hours spent in an OFF state and daily duration of mild-to-moderate or severe peak-dose dyskinesia. Prespecified primary continuous endpoints were the mean daily hours spent in an OFF state and the daily duration of mild-to-moderate or severe peak-dose dyskinesia. These parameters were quantified using standardized 24 h PD motor diaries completed by patients and caregivers for three consecutive days prior to each clinical visit, supplemented by retrospective clinician interview based on the MDS-UPDRS Part IV framework. The same measurement methodology was strictly applied across all longitudinal milestones. Prespecified secondary endpoints included global neurological staging determined using the Hoehn and Yahr (H&Y) scale, the prevalence of binary complications (such as early morning akinesia, sudden OFF, end-of-dose dystonia, freezing of gait) and tracked adjunctive oral medication classes. Cognitive baseline was verified using MMSE. Safety information was obtained at each scheduled assessment and included adverse-event type, severity, suspected relationship to LECIG, infusion hardware, action taken, resolution status, and treatment discontinuation. Deaths were reviewed by the treating multidisciplinary team based on the clinical documentation.

### 2.4. Statistical Analysis

Statistical analysis was performed using Prism 8.4.1 (GraphPad Software, San Diego, CA, USA). Continuous variables were expressed as mean ± standard deviation (SD) or as mean differences with 95% confidence intervals (95% CI) where applicable. Data distribution and normality were evaluated using Shapiro–Wilk tests and visual inspection of Q–Q plots. Agreement between the formula-derived initial estimates and the final clinically optimized flow-rate was assessed using Bland–Altman analysis, with percentage differences calculated as 100 × (B − A)/[(A + B)/2], where “A” represented the initial calculated estimate and “B” the final optimized value; the mean percentage difference and 95% limits of agreement are reported. For continuous duration metrics of specific motor complications (e.g., dyskinesia hours), means were calculated exclusively among the sub-group of patients actively experiencing the symptom at that milestone, rather than averaging across the entire cohort.

To account for repeated-measures correlations across all milestones in the cohort, an internally generated total clinical disease burden score was calculated as OFF time (hours/24 h) + severe peak-dose dyskinesia time (hours/24 h) + 0.5 × mild/moderate dyskinesia time (hours/24 h) + binary complication count, where the binary complication count added 1 point for each of the following if present: early morning akinesia, freezing of gait, end-of-dose dystonia, and sudden OFF episodes. Higher scores indicate greater overall clinical burden. In this cohort, the theoretical range was approximately 0–28 continuous points. This composite was exploratory, internally generated, and not externally validated; it was not intended to replace established clinical outcomes. The score was analyzed using a linear mixed-effects model (LMM) with a random intercept for each patient, fitted via Restricted Maximum Likelihood (REML) and assuming an unstructured residual covariance. Post hoc pairwise comparisons against baseline were adjusted using the Holm–Šídak method [39,40].

To accommodate the repeated-measures structure and appropriately handle missing longitudinal data (e.g., intercurrent deaths or dropouts) under a missing-at-random (MAR) assumption without imputation, continuous longitudinal parameters (daily OFF time, mild/moderate dyskinesia hours, Hoehn & Yahr staging, daily levodopa dose, and pump flow rates) were analyzed using LMM. Models were fitted via REML and included a random intercept for each subject, assuming an unstructured residual covariance. Baseline heterogeneity, disease duration, and previous oral treatment were not included as covariates or interaction terms in this exploratory model. Post hoc pairwise comparisons between baseline and subsequent follow-up milestones (initiation, 6, 12, and 18 months) were adjusted for multiple comparisons using the Holm–Šídak method.

For non-parametric repeated-measures ordinal parameters or zero-inflated continuous variables where model residuals violated normality assumptions (e.g., severe dyskinesia duration), the Friedman test was used, followed by Dunn’s post hoc test for multiple comparisons. Longitudinal changes in binary motor complication prevalence (e.g., early morning akinesia, freezing of gait) across all five milestones were evaluated using Cochran’s Q test and Generalized Estimating Equations (GEE) [41] with a binomial logit link function. Changes in categorical medication usage from baseline to 18 months were analyzed using paired *t*-tests and McNemar’s tests. All tests were two-tailed, and statistical significance was defined as *p* < 0.05. While post hoc multiplicity corrections (Holm-Šídák and Dunn’s tests) were rigorously applied to control Type I error across the multiple longitudinal timepoints within individual continuous and ordinal models, no global family-wise error rate correction was applied across the broader panel of distinct secondary clinical endpoints (e.g., individual medication classes, specific binary motor symptoms). Consequently, *p*-values reported for secondary and exploratory endpoints are nominal and should be interpreted as hypothesis-generating rather than definitive.

Missing data handling and cohort denominators. Longitudinal disposition was tracked at each milestone (*N* = 41 at baseline and initiation; *n* = 40 at 6 months; *n* = 39 at 12 months; *n* = 38 at 18 months). LMM and GEE incorporated all available longitudinal observations across all enrolled subjects (*N* = 41), leveraging REML and GEE mechanisms to accommodate MAR data without artificial imputation. For descriptive cross-sectional proportions and end-point paired comparisons (McNemar tests, paired *t*-tests) at 18 months, denominators represent evaluable patients with data at the relevant visit(s).

## 3. Results

### 3.1. Cohort Baseline Characteristics

The longitudinal study cohort comprised 41 consecutive advanced PD patients (23 males, 56.1%; 18 females, 43.9%, see Table 1). Mean age was 65.39 ± 8.32 years, with mean disease duration of 10.46 ± 4.23 years. Baseline MMSE score was 27.24 ± 1.99. Prior to LECIG initiation, patients took an average of 5.32 ± 0.52 levodopa doses daily (mean baseline oral levodopa dose 834.1 ± 244.1 mg/day).

Adjunctive therapy was widespread: 33 patients (80.5%) were on dopamine agonists (pramipexole: *n* = 7, mean 2.25 ± 0.73 mg/day; ropinirole: *n* = 10, mean 12.0 ± 5.66 mg/day; rotigotine: *n* = 16, mean 8.59 ± 2.09 mg/day); 32 patients (78.0%) used entacapone; 26 patients (63.4%) were on rasagiline; and 12 patients (29.3%) received amantadine. Regimen mapping showed quadruple therapy (LD + DAs + COMTi + MAOBi) was most common (*n* = 17, 41.5%), followed by triple therapy (LD + DAs + COMTi, *n* = 11, 26.8%). Baseline mean OFF time was 4.67 ± 0.79 h/day. Mild/moderate dyskinesia was present in 26 patients (mean duration among affected patients: 2.75 h/day). Severe dyskinesia affected 9 patients (mean duration among affected patients: 2.17 h/day). Baseline H&Y staging was 3.17 ± 0.38 in ON state and 4.24 ± 0.43 in OFF state.

### 3.2. Titration Kinetics and Dosing Dynamics

The mean inpatient titration duration required to achieve optimal motor stabilization was 5.58 ± 1.50 days (median 6.00 days). At initiation, the mean daily continuous infusion duration was programmed to 17.46 ± 1.42 h/day (median 18.00 h). The real optimized pump parameters delivered a total continuous gel volume of 50.07 ± 17.16 mL/day, utilizing a mean morning bolus of 6.12 ± 1.31 mL and mean extra rescue doses of 1.62 ± 0.64 mL/infusion. This optimized gel regimen yielded a real levodopa dose at initiation of 1247 ± 401.3 mg/day.

To evaluate the predictive accuracy of standard pre-switch dosing formulas, initial theoretical pump settings derived from baseline LEDD (calculated mean 1327 ± 288.9 mg/day) were formally compared against final inpatient initiation settings.

The initial continuous flow rate estimated from pre-switch LEDD was 2.38 ± 0.54 mL/h. Following inpatient fine-tuning, the real required continuous flow rate at initiation was significantly higher at 2.86 ± 0.88 mL/h. A paired *t*-test confirmed that this positive dose escalation from initial estimate to discharge was statistically significant (*p* = 0.0035). Correlation and linear regression analysis demonstrated virtually no predictive relationship between the initial calculated flow rate and the final discharge flow rate (*p* = 0.5851, r^2^ = 0.0077), indicating that initial LEDD calculation explained less than 1% (0.77%) of the variance in final discharge continuous pump settings. Also, comparing overall cohort averages, there was no statistically significant difference between baseline calculated LEDD (1327 ± 288.9 mg/day) and the real levodopa dose administered at discharge (1247 ± 401.3 mg/day; paired *t*-test, *p* = 0.2629). However, despite this group-level alignment, correlation and linear regression analysis demonstrated poor predictive value on an individual patient basis (*p* = 0.2800, r^2^ = 0.023). Pre-switch LEDD accounted for only 2.3% of the observed individual-level variance in real discharge levodopa requirements. Bland–Altman analysis demonstrated substantial individual-level variability between the formula-derived estimates and the final optimized settings. For flow rate, the mean percentage difference was 15.14%, with 95% limits of agreement from −63.94% to 94.22%. For total daily levodopa dose, the mean percentage difference was −8.43%, with 95% limits of agreement from −78.69% to 61.83%. Thus, although the group-level estimates were broadly similar, the formula-derived values did not reliably predict the final individualized maintenance requirements.

### 3.3. Longitudinal Evaluation of Motor Parameters (Table 2)

**Figure 1:**
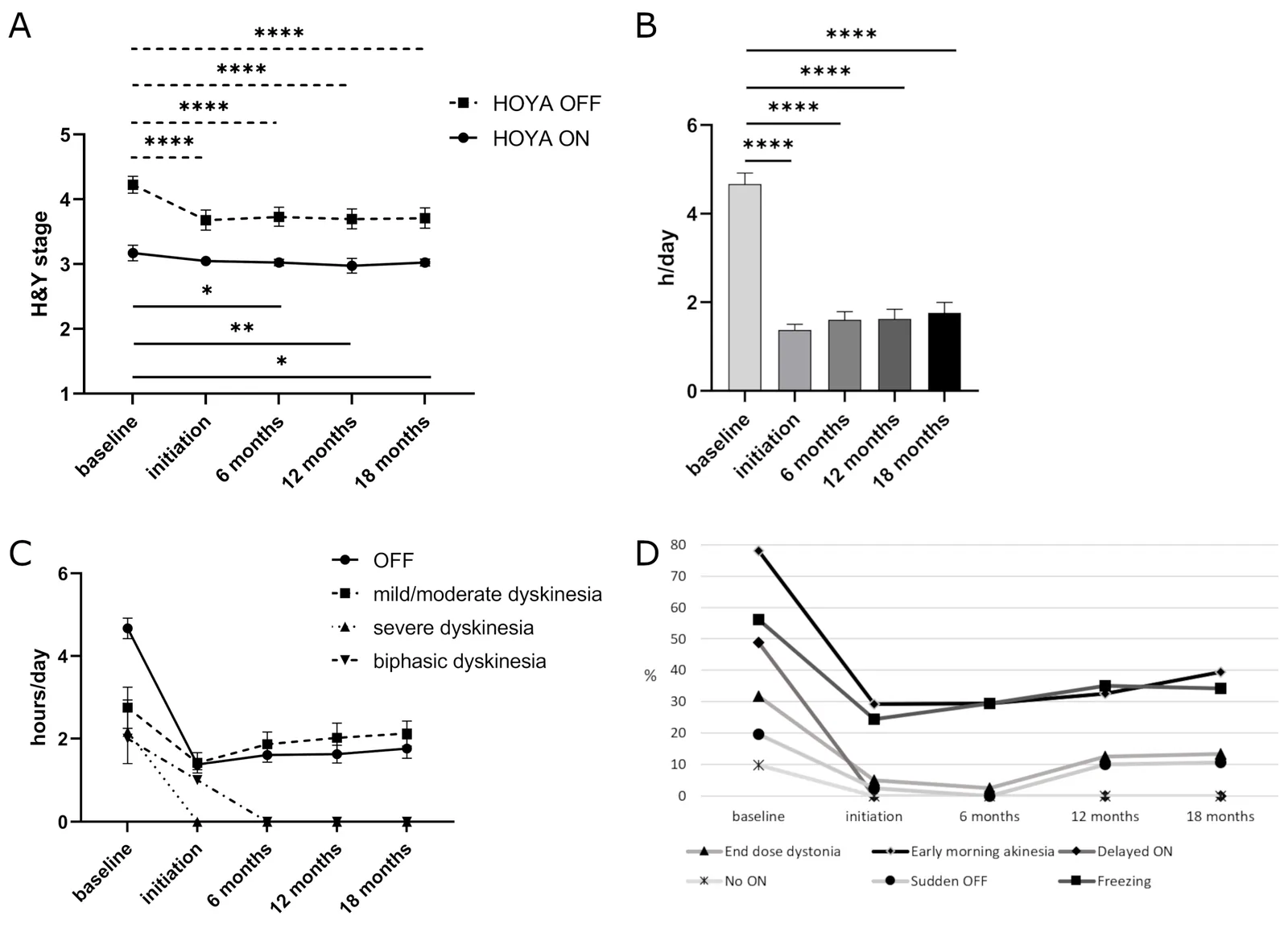
Change in motor parameters during the observation period. (**A**) Hoehn and Yahr stages. (**B**) OFF duration per day. (**C**) Clinical evolution of motor complications. (**D**) Longitudinal evolution of binary motor complications. * *p* < 0.05, ** *p* < 0.01, **** *p* < 0.0001.

Linear Mixed-Effects modeling demonstrated a rapid and sustained reduction in continuous daily OFF time from baseline (4.67 ± 0.79 h) to 1.55 ± 0.69 h at 18 months (LMM main effect *p* < 0.0001; mean difference at 18 months: −3.12 h, 95% CI: −3.42 to −2.82; Holm-Šídák adjusted *p* < 0.0001, Figure 1B). Severe peak-dose dyskinesia was completely eliminated at initiation and remained absent across all follow-up milestones (Friedman *p* < 0.001; Dunn’s post hoc *p* < 0.001 across all visits). Mild-to-moderate dyskinesia exhibited a non-linear trajectory; following an initial decline at initiation (1.42 ± 0.58 h), it gradually plateaued at 18 months (2.13 ± 0.65 h; LMM *p* < 0.0001), remaining significantly below baseline levels (mean difference: −0.62 h, 95% CI: −0.98 to −0.26; adjusted *p* = 0.0008, Figure 1C).

Hoehn and Yahr staging in the OFF phase improved from 4.24 ± 0.43 at baseline to 3.74 ± 0.45 at 18 months (LMM *p* < 0.0001; mean difference: −0.50, 95% CI: −0.66 to −0.34; adjusted *p* < 0.0001, Figure 1A). Categorical stage distribution analysis of the 38 evaluable patients at 18 months demonstrated that while the ON-state shift is statistically significant (*p* = 0.0176), the absolute numerical change (−0.14 points) is minor. Only 15.8% of patients (*n* = 6) experienced a discrete 1.0-stage improvement in the ON state, while 84.2% (*n* = 32) remained stable. Conversely, the continuous OFF-state reduction of −0.50 points translated to substantial categorical shifts: 47.4% (*n* = 18) of patients improved by at least one full stage (17 by 1.0 stage, 1 by 2.0 stages), and 52.6% (*n* = 20) remained stable, with zero patients worsening in either state.

No significant longitudinal shifts were observed in overall real levodopa daily dose (*p* = 0.2629) or pump flow rates (*p* = 0.7812) across maintenance visits.

**Table 2:** Longitudinal stabilization of motor parameters *.

Parameter	Baseline	Initiation	6 Months	12 Months	18 Months	Net Change at 18 Months (95% CI)	*p*-Value
Mean OFF (h/24 h)	4.67 ± 0.79	1.34 ± 0.40	1.61 ± 0.56	1.55 ± 0.67	1.55 ± 0.69	−3.12 (−3.42 to −2.82)	*p* < 0.0001 ^†^
Mild/moderate dyskinesia	2.75 ± 1.23, *n* = 26	1.42 ± 0.58, *n* = 24	1.87 ± 0.68, *n* = 23	2.02 ± 0.82, *n* = 23	2.13 ± 0.65, *n* = 20	−0.62 (−0.98 to −0.26)	*p* < 0.0001 ^†^
Severe dyskinesia	2.17 ± 1.00, *n* = 9	0.00	0.00	0.00	0.00	−2.17 (−2.48 to −1.86)	*p* < 0.001 ^†^
Real Levodopa (mg/d)	1327 ± 288.9	1247 ± 401	1174 ± 381	1321 ± 436	1359 ± 485	+32.0 (−135.0 to +199.0)	*p* > 0.05
Flow rate (mL/h)	N/A	2.86 ± 0.88	2.88 ± 0.89	2.85 ± 0.85	2.81 ± 0.88	−0.05 (−0.18 to +0.08) ^‡^	*p* > 0.05
H&Y Scale, ON	3.17 ± 0.38	3.05 ± 0.22	3.02 ± 0.16	2.98 ± 0.36	3.03 ± 0.16	−0.14 (−0.26 to −0.02) Imp: 15.8%, Stab: 84.2%	*p* = 0.0176 ^†^
H&Y Scale, OFF	4.24 ± 0.43	3.71 ± 0.46	3.76 ± 0.43	3.73 ± 0.45	3.74 ± 0.45	−0.50 (−0.66 to −0.34) Imp: 47.4%, Stab: 52.6%	*p* < 0.0001 ^†^

Data presented as mean ± standard deviation. Abbreviations: CI, Confidence Interval; H&Y, Hoehn and Yahr stage; h/24 h, hours per 24 h; Imp, improved; Stab, stable (see Section 3.3 for further description). * Analyzed via LMM fitted with REML and subject-specific random intercepts. *p*-values reflect omnibus main effect across time; pairwise post hoc comparisons against baseline were adjusted using the Holm-Šídák multiplicity correction. ^†^ Zero-inflated continuous outcome analyzed via non-parametric Friedman test with Dunn’s post hoc correction. ^‡^ Change calculated from initiation baseline flow rate setting.

### 3.4. Longitudinal Evolution of Binary Motor Complications (Table 3, Figure 1D)

Early morning akinesia dropped from 78.0% to 36.6% at 18 M (*p* < 0.001). Sudden OFF states dropped from 19.5% to 9.8% (*p* < 0.001). End-of-dose dystonia fell from 31.7% to 12.2% (*p* = 0.002). Freezing of gait dropped from 56.1% (23/41) to 31.6% (12/38 completers; Cochran’s Q *p* < 0.001, GEE *p* = 0.001).

**Table 3:** Prevalence of binary motor complications across longitudinal milestones.

Symptom	Baseline	Initiation	6 Months	12 Months	18 Months	Cochran’s Q	GEE *p*-Value
Early morning akinesia	78.0%	29.3%	29.3%	31.7%	36.6%	*p* < 0.001	*p* < 0.001
End dose dystonia	31.7%	4.9%	2.4%	12.2%	12.2%	*p* < 0.001	*p* = 0.002
Sudden OFF	19.5%	2.4%	0.0%	9.8%	9.8%	*p* = 0.002	*p* < 0.001
Freezing of gait	56.1%	24.4%	29.3%	34.1%	31.6%	*p* < 0.001	*p* = 0.001

### 3.5. LMM Modeling of Total Clinical Disease Burden

The exploratory total clinical disease burden score decreased from a mean baseline of 9.33 (Table 4) to 2.78 at LECIG initiation and 3.49 at 18 months. In the linear mixed-effects analysis, scores were lower at each post-initiation assessment compared with baseline; the estimated difference at 18 months was −5.84 points (95% CI [−6.39, −5.15]; *p* < 0.001). Because this composite was internally generated and non-validated, these findings should be interpreted as an exploratory summary of motor complication burden rather than as evidence of a validated global disease-burden measure.

### 3.6. Adjunctive Polypharmacy Transitions

Dopamine agonist prevalence dropped significantly from 80.5% (33/41) at baseline to 42.1% (16/38) at 18 months (−38.4% relative decrease; McNemar *p* < 0.0001). Mean oral adjunct burden per patient dropped from 1.73 to 1.39 medications/patient (paired *t*-test, *p* = 0.0284). MAO-B inhibitor usage remained stable (63.4% to 53.7%, *p* = 0.2200). Amantadine increased slightly (29.3% to 36.8%, *p* = 0.3750) to manage mild dyskinesias without re-introducing oral agonists (Table 5).

### 3.7. Safety and Attrition

Zero patients discontinued therapy due to adverse events or hardware failure. During follow-up, one patient voluntarily discontinued LECIG at 12 months because of personal preference. Two additional patients died from sudden cardiac arrest: one at approximately 6 months and one before the scheduled 18-month assessment. Based on the documented circumstances of death, the presence of severe pre-existing cardiovascular disease, review of the clinical records, and multidisciplinary clinical assessment, the deaths were judged unlikely to be related to LECIG therapy, the infusion hardware, or the PEG-J procedure. Thus, 38/41 patients (92.7%) completed the planned 18-month assessment, but the treatment-retention proportion was 40/41 (97.6%), if unrelated deaths are excluded from treatment-discontinuation analyses. Among 18-month completers (*n* = 38), overall therapy retention remained high. Treatment-emergent AEs included weight loss (*n* = 8), mild non-progressive polyneuropathy (*n* = 2), depression/anxiety (*n* = 4), hypersexuality (*n* = 1), sialorrhea (*n* = 1), and stoma granuloma (*n* = 2).

## 4. Discussion

PD should be defined not as a single entity but as a clinical syndrome characterized by diverse phenotypic expressions and heterogeneous progression rates [42]. The clinical course of PD is typically progressive, although the pattern of progression can be highly variable. As the disease advances, therapeutic response becomes less predictable [43]. The clinical heterogeneity of PD is marked by a wide variation in symptoms, response to dopaminergic therapy, and the emergence of complex motor and non-motor complications [44,45].

The results of this 18-month longitudinal study provide strong, real-world evidence of the clinical efficacy, long-term durability, and polypharmacy management benefits of levodopa–entacapone–carbidopa intestinal gel infusion in patients with advanced Parkinson’s disease. By following a consecutive cohort across five systematic milestones from inpatient titration through a year and a half of home maintenance, our findings show that the initiation of continuous enteral dopaminergic stimulation was followed by an immediate and sustained reduction in several disabling motor complications that characterize advanced stages of the disease, while being associated with a significant rationalization and simplification of complex oral drug regimens.

### 4.1. Sustained Reduction in Daily OFF Time and Motor Stabilization

The most compelling primary outcome of our study is the rapid and sustained reduction in daily OFF hours, which dropped by 66.8% from the baseline at the 18-month endpoint. This therapeutic benefit was established immediately during the inpatient titration phase and remained remarkably stable across all subsequent long-term milestones. In the framework of existing literature, this reduction represents a substantial clinical improvement that is generally consistent with the historical benchmarks observed with traditional device-aided therapies. For instance, the landmark GLORIA registry, which evaluated standard levodopa-carbidopa intestinal gel in a large real-world cohort, reported a mean daily OFF time reduction of 3.9 h over 24 months [31]. Similarly, the COSMOS registry documented an average decrease of approximately 4.1 h in daily OFF states [46,47]. Our observed absolute net reduction of 3.12 h at 18 months firmly positions LECIG as a comparably powerful continuous infusion modality, despite our patients receiving lower cumulative amounts of jejunal levodopa per hour.

The pattern observed in our cohort is consistent with findings from recent real-world evaluations of LECIG across European movement disorder centers. In a single-center study of 30 advanced PD patients by Viljaharju et al., LECIG infusion led to an immediate, significant drop in daily OFF time that was fully maintained over long-term follow-up [23]. Furthermore, the interim analysis of the global ELEGANCE study demonstrated that the continuous delivery of the triple-combination gel provides immediate and highly reproducible motor stabilization [48,49]. The physiological mechanism driving this robust stabilization is the direct bypass of gastric transport [50]. Advanced PD is heavily complicated by gastroparesis, delayed gastric emptying, and erratic intestinal transport, which render oral drug absorption highly unpredictable and pulsatile [12,51]. By delivering levodopa directly into the proximal jejunum, LECIG may reduce the effect of gastrointestinal dysmotility, and may contribute to a more stable dopaminergic exposure [18,49]. This is clinically reflected in our cohort by the total elimination of delayed ON state and no ON phenomena, which remained completely cleared through 18 months of follow-up. The observed reduction in unpredictable motor fluctuations may reflect a more stable drug delivery and represents a clinically relevant improvement in patients’ daily predictability, matching the practical guidance outlined by Nyholm and Jost [18].

While this study reports a statistically significant reduction in continuous mean H&Y ON-state scores, categorical analysis confirms this does not represent a transformative clinical shift, as only 15.8% of patients achieved a discrete stage reduction. However, the H&Y OFF-state reduction (−0.50 points) proved highly clinically relevant. Nearly half of the cohort (47.4%) achieved at least a full-stage categorical improvement during OFF periods, representing a tangible restoration of mobility and functional independence when patients are at their most vulnerable.

### 4.2. Limited Predictive Value of Pre-Switch LEDD Estimates and the Role of Clinical Titration

A critical methodological insight from our 41-patient dataset is the limited predictive validity of standard pre-switch LEDD conversion algorithms [18,35]. In clinical practice, clinicians routinely calculate an initial continuous hourly flow rate based on pre-existing oral LEDD. In our cohort, while group-level averages showed no significant divergence between pre-switch calculated LEDD and real initiation levodopa intake, regression modeling revealed that baseline LEDD explained only 2.3% of individual variance (r^2^ = 0.023). Furthermore, patients required a significantly higher continuous flow rate at initiation than primarily calculated (2.86 mL/h vs. 2.38 mL/h, *p* = 0.0035), with pre-switch LEDD explaining less than 1% (r^2^ = 0.0077) of final continuous pump speed.

This dissociation may reflect pharmacokinetic and physiological differences between oral therapy and continuous enteral gel infusion [18]. Pre-switch oral LEDD reflects erratic gastrointestinal absorption, delayed gastric emptying, and variable peripheral enzymatic metabolism. Once continuous intrajejunal delivery is established with co-administered entacapone, peripheral levodopa bioavailability expands by 20% to 25%, while erratic absorption spikes are eliminated [22,26]. Consequently, individual requirements may also depend on postsynaptic receptor sensitivity, disease duration, daily waking hours, and other patient-specific factors not captured by pre-switch oral LEDD. These findings suggest that pre-switch LEDD calculations may provide a useful initial framework for pump programming but should not be regarded as definitive predictors of the final individualized maintenance settings established after clinical titration [18]. Instead, individual inpatient fine-tuning over a temporary nasojejunal testing phase (mean 5.58 days in our cohort) remains clinically important to optimize motor control and minimize the risk of under- or over-dosing [18,26].

### 4.3. Reorganization of Dyskinesia Severity and Neurodegenerative Progression

A notable finding from our longitudinal data is the structural reorganization of dyskinesia severity. Troublesome, severe peak-dose dyskinesias and biphasic dyskinesias were completely eliminated at initiation and remained entirely absent throughout the 18-month follow-up period. This stands in sharp contrast to conventional oral therapies, where narrowing therapeutic windows and pulsatile receptor stimulation inevitably exacerbate severe, disabling involuntary movements [6,8]. The continuous infusion model may reduce the sharp plasma concentration fluctuations that are thought to contribute to severe peak-dose dyskinesia, establishing a highly controlled therapeutic window [18].

This gradual re-emergence of mild involuntary movements may reflect the natural progression of central neurodegeneration. As Parkinson’s disease advances, progressive loss of striatal dopaminergic neurons and central buffering capacity can lead to a hypersensitive receptor profile. However, because this is an observational dataset, causality cannot be definitively established. Other contributing factors, such as longitudinal dose adjustments, modifications to adjunctive oral medications, survivor effects within the cohort, and inherent measurement variability, must be considered as plausible alternative explanations for this gradual expansion in mild dyskinesia duration.

Conversely, mild-to-moderate peak-dose dyskinesia exhibited a highly distinct, non-linear, biphasic trajectory. Following an initial sharp decline from baseline to initiation, it demonstrated a gradual, incremental increase over time. Although this duration trended upward at later milestones, it remained 40.6% below baseline values. This gradual re-emergence of mild involuntary movements may reflect the natural, progression of central neurodegeneration [52]. As Parkinson’s disease advances, there is a progressive loss of striatal dopaminergic neurons, leading eventually to a depletion of central buffering and storage capacity, alongside a hypersensitive and hyper-reactive dopamine receptor profile [8]. Consequently, even under highly stable plasma concentrations, the central motor network becomes increasingly sensitive to dopaminergic stimulation, manifesting as an expansion of mild dyskinesia duration. Crucially, however, because LECIG provides a more continuous delivery pattern than oral medication, these re-emerging involuntary movements can remain strictly mild and non-troublesome. They did not cause distress or functional impairment, while daily activities and independence appeared to remain preserved in the observed cohort. This specific biphasic phenomenon is consistent with initial clinical experiences reported by Öthman et al. [46] and long-term observations in the ELEGANCE [48,49] and the COSMOS [53,54] studies, where long-term continuous infusion was associated with a shift toward a more manageable, non-disabling clinical dyskinesia phenotype rather than eliminating it entirely. However, because this is an observational dataset, causality cannot be definitively established. Other contributing factors, such as longitudinal dose adjustments, modifications to adjunctive oral medications, survivor effects within the cohort, and inherent measurement variability, must be considered as plausible alternative explanations for this gradual expansion in mild dyskinesia duration.

### 4.4. Reduction in Complex Motor Complications

Beyond standard OFF and ON metrics, our study tracked complex secondary motor complications that heavily compromise patient independence and increase caregiver burden. Early morning akinesia, which affected 78.0% of our cohort at baseline, fell to 29.3% at initiation and remained markedly suppressed at 36.6% at 18 months. This substantial reduction was associated with the initiation of the continuous infusion paradigm and the use of an optimized morning bolus regimen, which rapidly overcomes nocturnal dopaminergic depletion and ensures a rapid transition to the ON state upon waking, bypassing the standard delayed absorption of oral waking doses [18]. Similarly, sudden unpredictable OFF states were heavily reduced, whereas end-of-dose dystonia was also highly responsive.

Freezing of gait, one of the most disabling and unpredictable symptoms of aPD and a leading risk factor for falls [55,56], dropped sharply from a baseline prevalence of 56.1% to 24.4% after initiation, and remained low through 18 months, affecting 31.7%. Freezing of gait in aPD often possesses a dopaminergic-responsive component, occurring primarily during wearing-OFF or sudden transitional states [57,58,59]. By smoothing out plasma volatility and maintaining stable, continuous central stimulation, LECIG may have contributed to a more stable locomotor function, and was associated with a lower prevalence of freezing episodes during follow-up. The consistency of the observed changes across follow-up was supported by the internally generated LMM constructed for the composite total clinical disease burden score. This exploratory score was lower after LECIG initiation and remained lower than baseline throughout the 18-month observation period. Also, the model estimated a patient-level random-intercept variance of 0.902. This variance component reflects between-patient variability in the modeled clinical-burden score but does not establish that the observed longitudinal changes were independent of baseline heterogeneity, disease duration, or previous oral treatment, as these variables were not modeled as covariates or interaction terms. The observed reduction in the composite score supports the longitudinal findings for the prespecified motor outcomes but does not establish treatment-response uniformity, causality, or permanence. Because the score was internally generated, incorporated weighted continuous and binary components, and was not externally validated, the composite analysis should be regarded as supportive and hypothesis-generating. The individual prespecified motor outcomes therefore remain the principal clinical findings. These observational findings support the clinical utility of LECIG as an effective advanced treatment option among DATs for aPD patients, though future head-to-head comparative studies are needed to establish optimal therapy sequencing [26,60,61].

### 4.5. Structured Rationalization of Adjunctive Polypharmacy

A major secondary objective of this study was to map the long-term evolution of adjunctive oral polypharmacy following the initiation of continuous enteral infusion. Advanced PD patients are frequently managed with highly complex multidrug regimens, combining multiple doses of levodopa with various adjunctive classes (DAs, MAO-Bi, COMTi, amantadine) in an attempt to prolong oral efficacy [62,63]. This polypharmacy substantially increases pill burden, compromises compliance, and significantly elevates the risk of severe neuropsychiatric and systemic side effects, such as impulse control disorders, hallucinations, daytime somnolence, and severe orthostatic hypotension [10,64]. In our cohort, following establishment of continuous dopaminergic delivery via LECIG, we observed an 18.3% net reduction in total adjunctive medication burden over 18 months, with the mean oral adjunct burden per patient falling significantly from 1.73 to 1.39 medications/patient.

The most clinically meaningful transition occurred within the dopamine agonist class, where active user prevalence fell from 80.5% at baseline to 42.1% at 18 months. Crucially, this substantial de-escalation achieved statistical significance, indicating that dopamine agonist use decreased during follow-up after initiation of continuous intestinal levodopa infusion. This association is compatible with a clinically guided reduction in adjunctive therapy but does not establish definitively that LECIG itself will permanently enable dopamine agonist withdrawal [26,64]. From a clinical perspective, successfully tapering and discontinuing dopamine agonists in over a third of baseline users represents an important observed change in treatment strategy. It simplifies daily regimens and may reduce exposure to the long-term neuropsychiatric and cardiovascular risks associated with high-dose agonist therapy [10,18]. Our data also revealed distinct trajectories among other adjunctive classes. The usage of monoamine oxidase B inhibitors (rasagiline) remained stable, which confirms that clinicians frequently retain MAO-Bi as a reliable, safe background maintenance therapy to support baseline dopaminergic tone [64]. Conversely, amantadine was the only therapeutic class to undergo a slight longitudinal increase (rising from 29.3% at baseline to 36.8% at 18 months, +7.5% absolute increase, McNemar *p* = 0.3750). This transition may reflect a deliberate, highly tailored clinical strategy: rather than re-introducing or escalating high-risk dopamine agonists to manage the gradual re-emergence of mild motor symptoms at later milestones, amantadine use increased modestly during follow-up, possibly reflecting individualized management of residual or re-emerging dyskinesias. Amantadine acts as an NMDA receptor antagonist that is uniquely effective at managing mild peak-dose dyskinesias and optimizing motor performance without compromising neuropsychiatric safety [65,66]. This pattern is consistent with individualized adjustment of adjunctive therapy following continuous infusion, although the observational design does not permit attribution of these changes specifically to LECIG.

### 4.6. Pharmacokinetic Synergy and Polyneuropathy Risk Mitigation

The integration of entacapone directly within the intestinal gel formulation provides a distinct, foundational pharmacological advantage over traditional levodopa-carbidopa intestinal gel [67]. In standard LCIG therapy, extending levodopa availability requires escalating the continuous infusion volume and rate, leading to high cumulative daily levodopa exposure [68,69]. In contrast, the addition of entacapone inhibits the peripheral COMT-mediated metabolism of levodopa, significantly increasing its plasma half-life and expanding its central bioavailability (AUC) by 20% to 25% [22]. This pharmacological combination has been reported to permit an approximate 20% to 35% reduction in the total daily continuous levodopa dose required to achieve equivalent or superior clinical control. This dose-reduction capability carries clinically relevant long-term safety benefits, particularly regarding the mitigation of peripheral neuropathy [70,71]. Prolonged exposure to high cumulative doses of traditional LCIG has been strongly linked to a high incidence of acute and subacute large-fiber and small-fiber polyneuropathy, a disabling complication that often limits long-term therapy [72]. The primary pathophysiological driver of this neuropathy is the accumulation of the toxic metabolite homocysteine, which is generated via the methylation pathway as levodopa is metabolized by COMT [18,73].

Because the entacapone component of LECIG directly blocks this peripheral methylation pathway, it may reduce levodopa-related homocysteine accumulation and may therefore have a potential long-term protective effect against axonal degradation [26,73]. It must be mentioned, however, that homocysteine concentrations and axonal outcomes were not directly assessed in this study. Nevertheless, the relatively low number of mild, non-progressive neuropathy cases observed in this cohort is compatible with a favorable safety profile, even if it does not prove a protective mechanism. Over 18 months of continuous tracking, treatment-emergent polyneuropathy was limited to just two cases. Crucially, these cases were mild, strictly large-fiber, remained entirely non-progressive, and did not impair standard activities of daily living or require treatment modification. This low incidence and benign trajectory stand in sharp contrast to historical LCIG data, where polyneuropathy rates frequently exceed 30% to 40% over similar tracking intervals [72,74]. These findings are consistent with a favorable observed long-term safety and tolerability of the triple-combination formulation, reinforcing its advantages in real-world clinical practice.

### 4.7. Long-Term Safety, Attrition, and Regional Socioeconomic Relevance

The overall safety and tolerability profile of LECIG in our study was favorable. All documented events (weight loss in 8 patients, mild polyneuropathy in 2 patients, depression/anxiety in 4 patients, hypersexuality in 1 patient, sialorrhea in 1 patient, stoma granuloma in 2 patients) were mild-to-moderate, non-severe, and completely manageable using standard conservative medical care. These findings are consistent with published clinical and real-world data, which generally describe LECIG as having a favorable and manageable safety profile, with predominantly mild-to-moderate device-, infusion-site-, or dopaminergic treatment-related adverse events and relatively low rates of permanent treatment discontinuation. However, the interpretation of this comparison should take into account differences in study populations, follow-up duration, patient-selection criteria, monitoring intensity, and adverse-event definitions across studies [18,26,27,75].

Most importantly, there was a single therapy discontinuation during the 18-month observation period because of personal preference; no discontinuation was attributed to an adverse event or hardware failure. Two patients died from sudden cardiac arrest. These patients possessed complex, severe pre-existing cardiovascular disease, and a rigorous multidisciplinary review concluded that the death was unrelated to the LECIG therapy or the infusion hardware. These data align our results with safety profiles established in large-scale registries such as the ELEGANCE study [49] and the GLORIA registry [31].

This high long-term retention rate indicates good patient compliance, comfort, and physical handling, which may reflect the smaller, lighter micro-infusion pump hardware, patient selection, clinical follow-up, or other unmeasured factors.

### 4.8. Regional Treatment Constraints and Clinical Applicability

Finally, the results of this study must be interpreted within the regional and socioeconomic context of our tertiary neuro-movement specialist center in Târgu Mureș, Romania. As detailed by Szász et al. [28], patient access to alternative device-aided therapies, such as deep brain stimulation (DBS) or subcutaneous apomorphine pumps, is severely constrained in our regional clinical environment due to deep structural, financial, and socioeconomic limitations [3,60]. Consequently, enteral gel infusion stands as the primary available advanced intervention for medication-refractory aPD in our region. Because of these constraints, regional clinicians routinely maximize oral and transdermal combinations to their absolute therapeutic limits before introducing an invasive surgical procedure, causing aPD cohorts in our region to present with exceptionally high baseline symptom severity, severe motor complications, and substantial adjunctive polypharmacy [37]. In this context, we would also like to point out that in accordance with the conclusions of our multidisciplinary team’s previous publications, the use of the available dopaminergic agents appears to be similar to the manner of use described in the literature [76,77,78].

The observed immediate and sustained improvements following LECIG initiation, alongside a successful reduction in pill burden, in such a highly severe, brittle, real-world cohort underscores its clinical versatility and robustness. These findings support further evaluation of LECIG as an advanced treatment pathway, capable of delivering significant clinical outcomes even in challenging, resource-constrained clinical settings.

## 5. Methodological Limitations

We acknowledge several key limitations inherent to this study. First, its retrospective chart-review design and open-label nature introduce potential risks of selection or reporting biases. Second, the absence of a parallel oral-therapy or traditional LCIG control arm limits our ability to make direct, randomized comparative assertions. Third, while this single-center cohort provides real-world longitudinal data on 41 patients, larger multi-center prospective registries are warranted to further generalize these findings. Fourth, while longitudinal milestone comparisons within individual models were statistically corrected, we did not apply a global multiplicity correction across the wide array of distinct secondary clinical and pharmacological endpoints; these nominal *p*-values should therefore be interpreted cautiously as exploratory. In addition, the uncontrolled observational design precludes definitive attribution of the observed clinical and medication changes to LECIG itself; secular disease changes, individualized clinical management, regression to the mean, and other unmeasured factors may have contributed.

Also, as homocysteine, vitamin B6, vitamin B12, folate, methylmalonic acid, and systematic electrophysiological parameters were not prospectively assessed, the proposed protective mechanism remains a pharmacological hypothesis supported by prior literature.

An additional limitation concerns the exploratory total clinical disease burden score. This internally generated composite has not been externally validated, and its components represent clinically distinct phenomena with potentially different functional importance. The weighting scheme described in the Methods reflects clinical judgment rather than empirical validation. Therefore, the score should be considered hypothesis-generating, and its apparent reduction should not be interpreted as evidence of a validated global treatment-response measure. Future prospective studies should use validated composite outcomes or prespecify and validate a clinically interpretable disease-burden instrument. In addition, the exploratory mixed-effects model did not include baseline severity, disease duration, or previous oral treatment as covariates or interaction terms; therefore, independence of the observed longitudinal changes from these characteristics was not assessed.

However, these limitations are strongly counterbalanced by the strengths of our methodology, which include consecutive patient recruitment, a complete multi-milestone longitudinal dataset extending to 18 months, and the application of highly robust statistical modeling (such as LMM with random intercepts per patient and GEE with binomial families) specifically designed to handle repeated-measures correlations. Our real-world data thus provide critical, real-life insights that successfully bridge the gap between tightly controlled clinical trials and routine neuro-movement specialist practice.

## 6. Conclusions

This 18-month longitudinal study demonstrates that levodopa–entacapone–carbidopa intestinal gel infusion was associated with immediate and sustained reductions in motor fluctuations, OFF time, and total clinical burden in 41 patients with advanced Parkinson’s disease. Severe dyskinetic manifestations were absent after initiation, and the prevalence of several complex motor complications decreased during follow-up. LMM supported a sustained reduction in the burden score across the available longitudinal observations.

In addition to long-term clinical efficacy, our data demonstrate that pre-switch oral LEDD calculations possess limited predictive value for individual final pump settings. Although group-level averages align, some patients require significantly higher continuous flow rates at discharge than mathematically projected. These findings indicate that pre-switch LEDD calculations may support initial pump programming but have limited individual-level ability to predict the final optimized maintenance settings; personalized inpatient titration therefore remains necessary.

Furthermore, LECIG initiation coincided with a structured reduction and simplification of adjunctive oral polypharmacy. This was accompanied by a statistically significant decrease in dopamine agonist use and total oral adjunct burden per patient, reducing pill burden and possibly mitigating long-term neuropsychiatric risks. The micro-infusion system was generally well tolerated, showing high long-term compliance and treatment retention. These real-world findings support the utility of LECIG as a viable advanced treatment option in specialized movement disorder centers, demonstrating an ability to simplify complex medical regimens and improve aPD management.

## Figures and Tables

**Table 1:** Baseline characteristics of the cohort.

Baseline Parameter (N = 41)	Value (Mean ± SD or *n* (%))
Age (years)	65.39 ± 8.32
Gender (Male/Female)	23/18 (56.1%/43.9%)
PD duration (years)	10.46 ± 4.23
MMSE score	27.24 ± 1.99
Baseline oral levodopa (mg/day)	834.1 ± 244.1
Baseline levodopa frequency	5.32 ± 0.52 doses/day
Dopamine agonist usage	33 (80.5%)
Entacapone prevalence	32 (78.0%)
Rasagiline prevalence	26 (63.4%)
Amantadine prevalence	12 (29.3%)
Quadruple therapy (LD + DA + COMTi + MAOBi)	17 (41.5%)
Daily OFF time (hours/24 h)	4.67 ± 0.79
Mild/Moderate dyskinesia (hours/24 h)	2.75 ± 1.23
Severe dyskinesia prevalence	9 (22.0%)
H&Y Staging (ON/OFF)	3.17 ± 0.38/4.24 ± 0.43

**Table 4:** LMM for total clinical disease burden score.

Variable Milestone	Coefficient	Std. Error	z-Value	*p*-Value	95% Conf. Interval
Intercept (baseline burden)	9.33	0.31	30.16	*p* < 0.001	[8.72, 9.94]
LECIG initiation milestone	−6.55	0.32	−20.64	*p* < 0.001	[−7.17, −5.93]
6-month milestone	−6.06	0.32	−19.11	*p* < 0.001	[−6.68, −5.44]
12-month milestone	−5.77	0.32	−18.18	*p* < 0.001	[−6.39, −5.15]
18-month milestone	−5.84	0.32	−18.42	*p* < 0.001	[−6.46, −5.22]

**Table 5:** Evolution of adjunctive oral medication classes across study milestones.

Medication Class/Metric	Baseline (N = 41)	Initiation (*n* = 41)	6 Months (*n* = 40)	12 Months (*n* = 39)	18 Months (*n* = 38)	Shift Δ	*p*-Value
DA prevalence	33 (80.5%)	26 (63.4%)	27 (67.5%)	23 (59.0%)	16 (42.1%)	−38.4%	McNemar *p* < 0.0001
MAO-Bi	26 (63.4%)	24 (58.5%)	23 (57.5%)	22 (56.4%)	20 (52.6%)	−10.8	McNemar *p* = 0.2200
Amantadine	12 (29.3%)	12 (29.3%)	14 (35.0%)	14 (35.9%)	14 (36.8%)	+7.5%	McNemar *p* = 0.3750
Mean adjunct burden (meds/pt)	1.73 ± 0.64	1.51 ± 0.59	1.60 ± 0.61	1.51 ± 0.60	1.39 ± 0.58	simpl.	Paired *t*-test *p* = 0.0284

Percentages at each milestone are calculated based on the number of active, evaluable patients (*n*) present at that specific follow-up visit.

## Data Availability

The original contributions presented in this study are included in the article. Further inquiries can be directed to the corresponding author.

## Abbreviations

The following abbreviations are used in this manuscript:

aPD	advanced Parkinson’s disease
AE/AEs	adverse events
COMTi	catechol-O-methyltransferase inhibitors
DA/DAs	dopamine agonists
DATs	device-aided therapies
GEE	Generalized Estimating Equations
H&Y	Hoehn and Yahr
LCIG	levodopa-carbidopa intestinal gel
LD	levodopa
LECIG	levodopa–entacapone–carbidopa intestinal gel
LEDD	Levodopa Daily Equivalent Dose
LMM	Linear Mixed-Effects Model
MAO-Bi	monoamine oxidase-B inhibitors
meds/pt	medications per patient
MMSE	Mini-Mental State Examination
PD	Parkinson’s disease
PEG-J	percutaneous endoscopic gastrojejunostomy
SD	standard deviation
simpl.	simplification
